# Supplementary material for: COVID-19 vaccination and Guillain-Barré syndrome: analyses using the National Immunoglobulin Database
Source: Brain. 2022 Feb 18;146(2):739–48. doi: 10.1093/brain/awac067 (PMC8903477; doi:10.1093/brain/awac067)
Supplement: awac067_Supplementary_Data [file awac067_supplementary_data.pdf]

BPNS/ABN COVID-19 Vaccine GBS Study Group

Hadi Manji, National Hospital of Neurology and Neurosurgery, Queen Square, University College London Hospitals NHS Foundation Trust, London

Tim Lavin, Manchester Centre for Clinical Neurosciences, Salford Royal NHS Foundation Trust, Manchester

James B. Lilleker, Manchester Centre for Clinical Neurosciences, Salford Royal NHS Foundation Trust, Manchester

David Gosal, Manchester Centre for Clinical Neurosciences, Salford Royal NHS Foundation Trust, Manchester

Robert D.M. Hadden, Kings College Hospital NHS Foundation Trust, London

Taylor Watson-Fargie, Institute of Neurological Sciences, Queen Elizabeth University Hospital, NHS Greater Glasgow and Clyde, Glasgow.

Kathryn Brennan, Institute of Neurological Sciences, Queen Elizabeth University Hospital, NHS Greater Glasgow and Clyde, Glasgow.

Andreas Themistocleous, Nuffield Department of Clinical Neurosciences, University of Oxford, Oxford, UK

Jacquie Deeb, Queens Hospital, Barking, Havering and Redbridge University Hospitals NHS Trust, Romford

Ana Romeiro, Wexham Park Hospital, NHS Frimley Health Foundation Trust, Slough

Puja R. Mehta, Kings College Hospital NHS Foundation Trust, London; UCL Queen Square Institute of Neurology, London

Dimitri Kullmann, National Hospital for Neurology and Neurosurgery, Queen Square, University College London Hospitals NHS Foundation Trust, London

James Miller, Royal Victoria Infirmary, The Newcastle upon Tyne Hospitals NHS Foundation Trust, Newcastle upon Tyne

Amar Elsaddig, Manchester Centre for Clinical Neurosciences, Salford Royal NHS Foundation Trust, Manchester

Adam Molyneux, John Radcliffe Hospital, Oxford University Hospitals NHS Foundation Trust, Oxford

Plamen Georgiev, Broomfield Hospital, Mid and South Essex NHS Foundation Trust, Chelmsford

Aaron Ben-Joseph, Maidstone Hospital, Maidstone and Tunbridge Wells NHS Trust, Kent

James Holt, The Walton Centre NHS Foundation Trust, Liverpool

Jacob Roelofs, Royal Victoria Infirmary, The Newcastle upon Tyne Hospitals NHS Foundation Trust, Newcastle upon Tyne

Fadi Alkufri, Kent and Canterbury Hospital, East Kent Hospitals University NHS Foundation Trust

David Allen, Southampton General Hospital, University Hospital Southampton NHS Foundation Trust, Southampton

Simon Shields, Somerset NHS Foundation Trust, Somerset

Stephen Murphy, Imperial College Healthcare NHS Trust, London

Harri Sivasathiaseelan, Homerton University Hospital NHS Foundation Trust, London

Richard Sylvester, Homerton University Hospital NHS Foundation Trust, London

Abdul Al-Saleh, The Royal Wolverhampton NHS Trust, Wolverhampton

Rhys Roberts, Addenbrookes Hospital, Cambridge University Hospitals NHS Foundation Trust, Cambridge

Kannan Nithi, Northampton General Hospital, Northampton General Hospital NHS Trust, Northampton

Lahiru Handdunnethi, John Radcliffe Hospital, Oxford University Hospitals NHS Foundation Trust, Oxford

Kate Wannop, Kings College Hospital NHS Foundation Trust, London

Amit Batla, Royal Free Hospital NHS Foundation Trust, London

Anna Sadnicka, Royal Free Hospital NHS Foundation Trust, London

Jananee Sivaganasundaram, Epsom and St Helier University Hospitals NHS Trust, Epsom

Tatyana Yermakova, Leeds General Infirmary, Leeds Teaching Hospitals NHS Trust, Leeds

Ravi Dasari, Kent and Canterbury Hospital, East Kent Hospitals University NHS Foundation Trust

Graziella Quattrocchi, North Middlesex Uniersity Hospital NHS Trust, London

Harriet Ball, North Bristol NHS Trust, Bristol

Rebecca Cooper, Princess Royal Hospital, Haywards Heath, University Hospitals Sussex NHS Foundation Trust

Daniel Whittam, Manchester Centre for Clinical Neurosciences, Salford Royal NHS Foundation Trust, Manchester

Mohanned Mustafa, Manchester Centre for Clinical Neurosciences, Salford Royal NHS Foundation Trust, Manchester

Gabriel Yiin, Great Western Hospitals NHS Foundation Trust, Swindon

Shayan Ashjaei, Kings College Hospital NHS Foundation Trust, London

Andrew J. Westwood, Tunbridge Wells Hospital, Maidstone and Tunbridge Wells NHS Trust, Kent

Michelle Dsouza, Kent and Canterbury Hospital, East Kent Hospitals University NHS Foundation Trust

Eng Chuan Foo, Calderdale Royal Hospital, Calderdale and Huddersfield NHS Foundation Trust, Halifax

Shwe Zin Tun, Calderdale Royal Hospital, Calderdale and Huddersfield NHS Foundation Trust, Halifax

Khine Khine Lwin, Calderdale Royal Hospital, Calderdale and Huddersfield NHS Foundation Trust, Halifax

Gorande Kanabar, East & North Hertfordshire NHS Trust
